# A matched-analysis on short-term and long-term (up to 5 years of follow-up) urinary incontinence outcomes after robot-assisted radical prostatectomy with and without anterior and posterior reconstruction: data on 1358 patients

**DOI:** 10.1007/s11255-023-03766-z

**Published:** 2023-08-30

**Authors:** Marco Rinaldi, Angelo Porreca, Sebastiano Di Lena, Luca Di Gianfrancesco, Michele Zazzara, Marcello Scarcia, Giuseppe Mario Ludovico

**Affiliations:** 1 Department of Oncological Urology, Veneto Institute of Oncology IOV-IRCCS, Padua, Italy; 2 Urology, Western Hospital Unit “San Pio”, Castellaneta, Italy; 3 Urology Department, General Regional Hospital F. Miulli, Acquaviva delle fonti (BA), Italy

**Keywords:** Prostate cancer, Robot-assisted radical prostatectomy, Anterior reconstruction, Posterior reconstruction, Continence, Urethral rhabdosphincter

## Abstract

**Purpose:**

We report a comparative monocentric study with a short and long-term follow-up with the aim to assess differences about urinary continence outcomes in patients treated with Robot-Assisted Radical Prostatectomy (RARP) with two different techniques: with anterior and posterior reconstruction and without any kind of reconstruction.

**Materials and methods:**

From January 2016 to September 2021, at the Department of Urology of the “F. Miulli” Hospital of Acquaviva delle Fonti, in Italy, 850 eligible patients underwent extraperitoneal RARP with anterior and posterior reconstruction and 508 without reconstructions.

**Results:**

In patients undergoing RARP with reconstructions 1 month after surgery the urinary continence was preserved in 287/850 patients (33.8%), 3 months after surgery in 688/850 (81%), 6 months in 721/850 (84.8%), 12 months in 734/850 (86.3%), 18 months in 671/754 (89%), 24 months in 696/754 (92.3%), 36 months in 596/662 (90%), 48 months in 394/421 (93.6%), 60 months in 207/212 (97.6%). In patients undergoing RARP without reconstruction 1 month after surgery urinary continence was preserved in 99/508 (19.4%), after 3 months in 276/508 (54.3%), 6 months in 305/508 (60%), 12 months in 329/508 (64.7%), 18 months in 300/456 (65.7%), 24 months in 295/456 (64.7%), 36 months in 268/371 (72.3%), 48 months in 181/224 (81%), 60 months in 93/103 (90.3%).

**Conclusion:**

In our case study, the RARP with anterior and posterior reconstruction technique is associated with a statistically significant higher rate (up to 48 months of follow-up) and a faster recovery of urinary continence compared to the technique without reconstructions.

## Introduction

The goal of radical prostatectomy (RP) with any approach is the eradication of prostate cancer and to preserve the function of the pelvic organs. The procedure involves the removal of the entire prostate with its intact capsule and seminal vesicles, followed by bladder-urethral anastomosis. Surgical approaches have evolved from open approaches to laparoscopic and robot-assisted techniques.

RP is indicated in case of localized intermediate-risk, in some cases of low-risk and high-risk or locally advanced PCa [1, 2].

The candidates for robot-assisted radical prostatectomy (RARP) are the same as for open surgery. It is possible to achieve wide surgical resection margins with robotic prostatectomy and an extended pelvic lymphadenectomy can be performed when desired [3].

RARP can be performed both extraperitoneally and transperitoneally and comparable results have been obtained for both approaches [4].

Bladder-urethral anastomosis can be performed with two semicontinuous sutures as described by Van Velthoven et al. [5].

The preservation of the integrity of the external urethral sphincter is crucial for post-RP continence. Less clear is the effect of reconstruction the surrounding supporting structures to return to continence. Several small randomized clinical trials were conducted, however, the analyses were hampered by variation in definitions of incontinence and surgical approach, such as open vs robotic and intraperitoneal vs extraperitoneal approach. In addition, the techniques used to perform both anterior and posterior reconstruction are varied. Anterior suspension is performed through the periosteum of the pubis or the combination of dorsal venous complex bound (DVC-bound) and puboprostatic ligaments (PPL). The posterior reconstruction of the rhabdosphincter is described on the Denonvilliers posterior fascia to the bladder, or on the posterior wall of the bladder.

The posterior reconstruction technique described by Rocco et al. [6, 7] for open prostatectomy consists of a two-step reconstruction, which involves the juxtaposition of the rahbdosphincter (urethral striated sphincter) to the residue of the Denonvilliers band (first step), followed by attachment of the median Rafe of the Denonvilliers fascia to the posterior portion of the bladder neck. The purpose of this reconstruction is to avoid caudal retraction of the sphincter complex, keeping the urethra in its anatomical and functional position in the pelvic floor.

Because there is contradictory evidence about the effectiveness of anterior and/or posterior reconstruction on the return to post-RP continence, currently there are not recommendations.

PCa treatment can affect a man both physically and mentally. The problems of patients with PCa all have an impact on the individual perception of quality of life (QoL) [8]. Psychological discomfort can be caused by the diagnosis of cancer, the symptoms of cancer, and/or the side effects of treatment. Taking QoL into account means relying on understanding patient values and preferences so that optimal treatment proposals can be formulated and discussed with the patient.

The second most common complication of RARP is long-term urinary incontinence [9, 10], but emptying urinary difficulties associated with bladder neck stenosis (1.1% after RARP) may also occur [11].

Systematic reviews documented complication rates after RARP [12–15] with average 12-month continence rates of 89–100%. A non-randomised controlled prospective study of patients undergoing RP in 14 centres showed that at 12 months after RARP 21.3% were incontinent.

There is not universally accepted definition of post-radical prostatectomy urinary continence: according to EAU guidelines, the definition of urinary continence after radical prostatectomy includes total control without loss or use of pads, loss of a few drops of urine without the use of pads, using one “safety” pad per day [16].

The causes of persistent incontinence after prostatectomy are multiple: sphincter deficiency, bladder abnormalities, including bladder hyperactivity, impaired compliance or contractility. Recent studies reported that post-prostatectomy incontinence is associated with internal sphincter deficiency in most cases, instead bladder dysfunction is rarely the cause [17].

There are several pre-operative predisposing factors for incontinence: especially, the risk of post-prostatectomy radical incontinence seems to be inversely proportional to the length of the membranous urethra and directly proportional to the patient’s age, prostate volume, BMI and ASA Score [18, 19].

In robotic surgery, other factors that affect continence recovery are the experience of the surgeon [20], bladder neck preservation and reconstruction techniques (anterior or posterior) [21]; in particular posterior musculo-fascial reconstruction seems to offer a slight advantage in terms of urinary continence recovery one month after surgery [22]. A further important independent predictive short-term continence recovery factor is the catheterization duration [23].

### Purpose of the study

The aim of this study was to re-evaluate patients undergoing extraperitoneal RARP for prostate cancer at the Department of Urology of the “F.Miulli” Hospital of Acquaviva delle Fonti, in Italy, during the period January 2016—September 2021, in order to verify the functional results about urinary continence in patients operated with two different approaches: with anterior and posterior reconstruction and without anterior and posterior reconstruction.

## Materials and methods

### Criteria for inclusion

In the period of January 2016—September 2021, 1461 procedures of extraperitoneal RARP were performed at the Department of Urology of the “F. Miulli” Hospital of Acquaviva delle Fonti, in Italy.

Among these procedures 859 were performed by a surgeon who used, for all cases, a technique of reconstruction both anterior and posterior and 602 from another surgeon who performed a procedure without anterior and posterior reconstruction.

Among the 859 patients who performed a technique with reconstruction 9 were treated with bladder neck plastic.

Among the 602 patients who did not undergo reconstruction, 94 underwent bladder neck plastic.

In order to make the two populations of patients homogeneous and comparable, those in which the bladder neck was not preserved were discarded from the population of the operated patients.

So of all these patients were eligible 850 patients (Mean age = 63.8 years; range = 70–40 years) treated with RARP with anterior and posterior reconstruction and 508 (Mean age = 65.8 years; range = 71–46 years) without any kind of reconstruction (Table 1).

**Table 1:** Age of the patients and intraoperative data of RARP with and without anterior and posterior reconstruction

	Age of the patients and intraoperative data of robot-assisted radical prostatectomy with anterior and posterior reconstruction	Age of the patients and intraoperative data of robot-assisted radical prostatectomy without anterior and posterior reconstruction
Mean age of the patients eligible	63.8 ± 6.8 SD range (70–40yy)	65.8 ± 6.3 SD range (71–46yy)
Average operating time	108.9 min	98.41 min
Bilateral nerve-sparing	717/850 (84.3%)	239/508 (47.1%)
Right nerve-sparing	12/850 (1.4%)	9/508 (1.8%)
Left nerve-sparing	7/850 (0.8%)	6/508 (1.2%)
Not nerve-sparing	114/850 (13.5%)	254/508 (49.9%)

### Surgical technique

The bladder-urethral anastomosis was performed with 2 “barbed” semicontinuous sutures V-Loc 3–0.

**Fig. 1:**
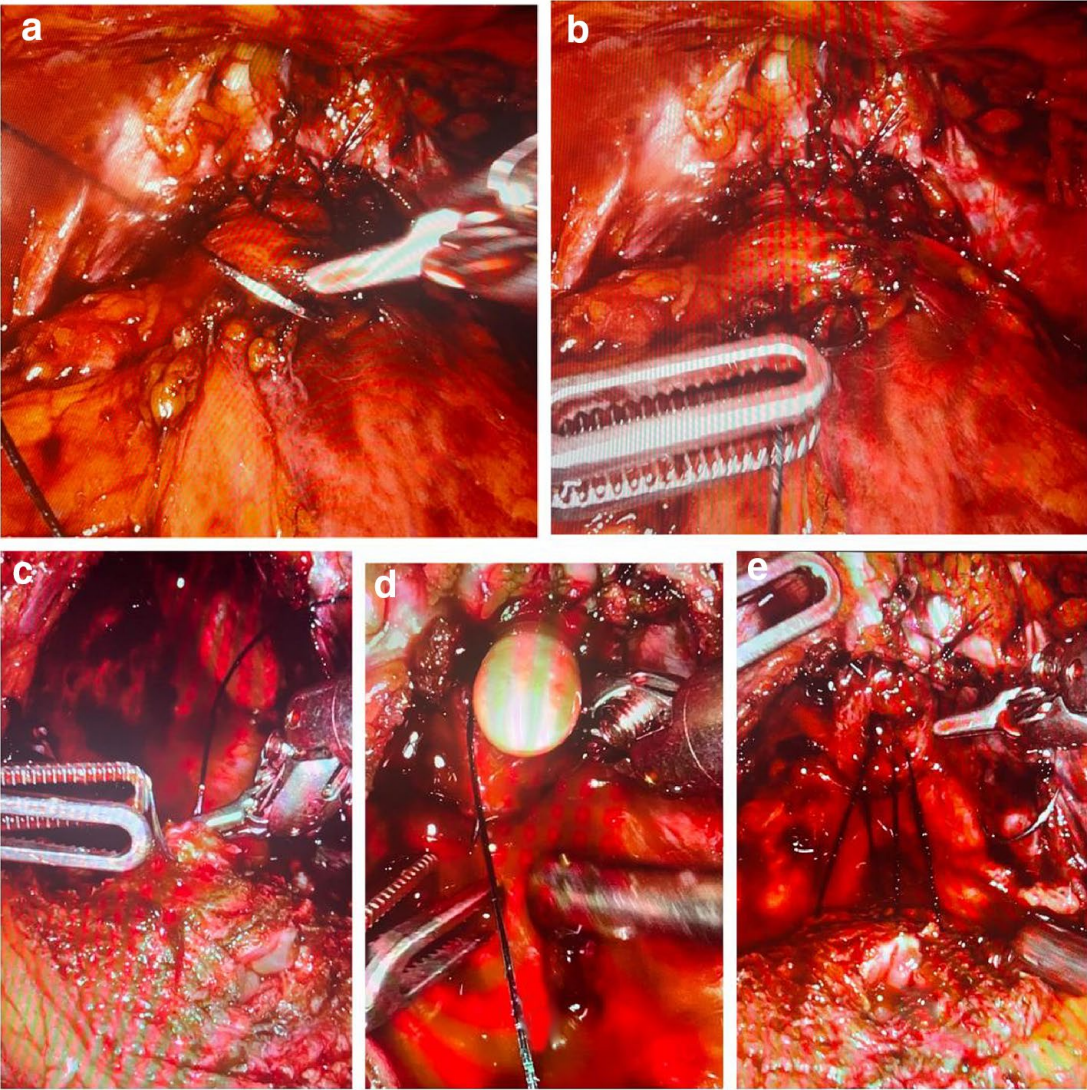
Anterior reconstruction (**a**, **b**) and posterior reconstruction (**c**, **d**, **e**)

The posterior reconstruction was performed with the same V-Loc “barbed” suture used for the anastomosis, which was passed on the rhabdosphincter (urethral sphincter striated) and on the Denonvilliers posterior fascia and the posterior wall of the bladder, just before performing anastomosis, instead the anterior reconstruction, with the same type of suture, at the end of the bladder-urethral anastomosis, passing it on the puboprostatic ligaments (PPL) and on the anterior wall of the bladder (Fig. 1).

### Peri-operative and post-operative management and follow-up

The mobilization of the patient generally took place in the first post-operative day.

The removal of the bladder catheter was scheduled to take place on the seventh day after surgery, with no cystographic control.

All patients undergoing RARP were trained to perform exercises aimed at the rehabilitation of the pelvic floor. Patients were instructed to properly contract the pelvic muscles and release the abdominal muscles. The practice at home included 45 contractions, in 3 sessions of 15 per day, progressively increased.

Patients treated with RARP underwent follow-up with a dosage of PSA one month after surgery, then every three months for the first two years and then every six months thereafter.

Patients were evaluated in follow-up with digital rectal exam (DRE), evaluation of total PSA values and evaluation of possible urinary incontinence or erectile disfunction, therefore also the occurrence of disease recurrence and the need of other treatments.

To objectively assess the presence and extent of possible urinary incontinence, we asked the patient about the number of pads used per day. In the case of no pads or the use of only a safety pad, the patient was considered continent, in the event that he declared that he had to change one or more pads per day, he was considered incontinent of variable size according to the number of pads/die.

Ultrasound evaluation of the post-urination residue was also performed in each patient.

### Statistical analysis

All patients were put into a database to undergo a retrospective cohort study.

Windows Excel for macOS was used to calculate mean values and to conduct other statistical analyses such as p values.

Up to 36 months p values less than 0,001 were considered statistically significant. At 48 months p value less than 0,005 and at 60 months p value less than 0,05 were considered statistically significant.

## Results

The average duration of RARP with anterior and posterior reconstruction was 108.9 min; the average duration of RARP without reconstructions was 98.4 min.

In the group with anterior and posterior reconstruction in 717/850 patients (84.3%) a bilateral nerve-sparing technique was performed, in 114/850 patients (13.5%) a non-nerve-sparing technique, in 12/850 (1.4%) a right unilateral nerve-sparing, in 7/850 (0.8%) a left unilateral nerve-sparing procedure.

In the group without reconstructions in 254/508 patients (49.9%) a non-nerve-sparing procedure was performed, in 239/508 patients (47.1%) a bilateral nerve-sparing technique, in 9/508 (1.8%) a right unilateral nerve-sparing, in 6/508 (1.2%) a left unilateral nerve-sparing (Table 1).

In the group with anterior and posterior reconstruction, definitive pathological staging showed a pT2a stage in 60/850 patients (4.3%), pT2c in 543/850 (63.9%), pT3a in 181/850 (21.3%) and pT3b in 66/850 (7.7%); the final Grade Group was 0 in 5/850 patients (0.6%), 1 in 327/850 (38,5%), 2 in 306/850 (35.9%), 3 in 147/850 (17.3%), 4 in 49/850 (5.8%) and 5 in 16/850 (1.9%); surgical margins were positive in 51/850 patients (6%).

In the group without anterior and posterior reconstruction, definitive pathological staging showed a pT2a stage in 45/508 patients (8.9%), pT2c in 294/508 (57.8%), pT3a in 124/508 (24.4%) and pT3b in 45/508 (8.9%); the final Grade Group was 0 in 11/508 patients (2.2%), 1 in 132/508 (26%), 2 in 177/508 (34.8%), 3 in 144/508 (28,3%), 4 in 33/508 (6.5%) and 5 in 11/508 (2.2%); surgical margins were positive in 91/508 patients (17.9%).

The duration of follow-up in patients undergoing RARP with and without anterior and posterior reconstruction was in the range of 12 to 60 months.

The short-term and long-term result that we considered in our study was urinary continence (defined as use of “no pad” or a “safety pad” per day).

In patients undergoing extraperitoneal RARP with anterior and posterior reconstruction 1 month after surgery the urinary continence was preserved in 287/850 patients (33,8%),after 3 months from surgery in 688/850 patients (81%), after 6 months in 721/850 (84,8%), after 12 months in 734/850 (86,3%), after 18 months in 671/754 (89%), after 24 months in 696/754 (92,3%), after 36 months in 596/662 (90%), after 48 months in 394/421 (93,6%), after 60 months in 207/212 (97,6%).

In patients undergoing extraperitoneal RARP without anterior and posterior reconstruction 1 month after surgery urinary continence was preserved in 99/508 (19,4%), after 3 months in 276/508 (54,3%), after 6 months in 305/508 (60%), after 12 months in 329/508 (64,7%), after 18 months in 300/456 (65,7%), after 24 months in 295/456 (64.7%), after 36 months in 268/371 (72.3%), after 48 months in 181/224 (81%), after 60 months in 93/103 (90.3%) (Table 2).

**Table 2:** Short-term and long-term functional results of robot-assisted radical prostatectomy

	RARP without reconstruction	RARP with anterior and posterior reconstruction	P value
Continent (0–1 safety pad) 1 month	99/508 (19.4%)	287/850 (33.8%)	0.00001
Continent (0–1 safety pad) 3 months	276/508 (54.3%)	688/850 (81%)	0.00000001
Continent (0–1 safety pad) 6 months	305/508 (60%)	721/850 (84.8%)	0.000000026
Continent (0–1 safety pad) 12 months	329/508 (64.7%)	734/850 (86.3%)	0.00000042
Continent (0–1 safety pad) 18 months	300/456 (65.7%)	671/754 (89%)	0.000000058
Continent (0–1 safety pad) 24 months	295/456 (64.7%)	696/754 (92.3%)	0.00000000037
Continent (0–1 safety pad) 36 months	268/371 (72.3%)	596/662 (90%)	0.00016
Continent (0–1 safety pad) 48 months	181/224 (81%)	394/421 (93.6%)	0.0049
Continent (0–1 safety pad) 60 months	93/103 (90.3%)	207/212 (97.6%)	0.054

## DISCUSSION

Currently there is no conclusive evidence on the effect of the reconstruction of the rhabdosphincter of the urethra for the return to urinary continence. Several RCTs were conducted whose limits were represented by the definition of incontinence and the type of surgical approach and also by the type of technique used for anterior and posterior reconstruction. There are currently no recommendations, but no studies have shown adverse oncological outcomes or complications with reconstruction.

Two studies showed no significant improvement by evaluating posterior reconstruction in RARP in the return to continence [24, 25]. Another RCT that studied the combination of anterior and posterior reconstruction found no improvement over a standard anastomosis without reconstruction [26].

Only the anterior suspension through the pubic periosteum in extraperitoneal RARP showed no advantage [27].

An RCT that evaluated anterior and posterior reconstruction in open retropubic radical prostatectomy (RRP) showed a significant improvement in the return to continence at one month and 3 months, but not at 6 months [28]. Another RCT that studied the front reconstruction plus the rear one using the Advanced Reconstruction of VesicoUrethral Support (ARVUS) technique and the strict definition of continence as “without pads”, showed a statistically significant improvement in continence to 2 weeks, 4 weeks, 8 weeks, 6 months and 12 months, compared to the reconstruction of Rocco back standard [29].

A meta-analysis, including ten studies about efficacy of total reconstruction vs non total reconstruction in patients treated with RARP or laparoscopic radical prostatectomy (using the definitions of urinary continence as 0–1 pad or 0 pad) revealed a significant improvement of return to urinary continence in favor of total reconstruction up to 24 weeks of follow-up with both the definitions of urinary continence and a significant up to 52 weeks only in the 0–1 safety pad subgroup [30].

Instead a cochrane review reported that RARP with posterior reconstruction alone improve urinary continence only 1 week after chateter removal but not thereafter [31].

There are not unique data, but the various studies suggest a possible early and long-term return to continence, but very long-term data is missing.

The prevalence of urinary incontinence after RARP is influenced by numerous factors, such as patient characteristics, surgeon experience, surgical technique, and methodological aspects, such as the definition of continence. In particular some studies used a definition of continence of “no pad” and others, more frequently, considered continents even patients using a “safety pad” per day.

In our case study, patients operated with extraperitoneal RARP technique in one arm were treated with anterior and posterior reconstructions technique and in the other with a no reconstruction technique: the goal of anterior and posterior reconstruction was to restore the length of the urethra-sphincter complex, prevent its retraction, avoid excessive tension of the vesico-urethral anastomosis and provide support to the urethral-sphincter complex. The technique is simple and reproducible, with a limited increase in operating time. It also helps to improve the support for the execution of the anastomosis.

Urinary continence in patients undergoing RARP with anterior and posterior reconstruction 1 month after surgery was preserved in 33.8% of cases, compared to 19.4% of patients not undergoing reconstruction, after 3 months with reconstruction in 81% of cases and in 54.3% without reconstruction, after 6 months preserved in 84.8% of cases with reconstruction and in 60% without reconstruction, after 12 months in 86.3% with reconstruction and in 64.7% without reconstruction, after 18 months in 89% with reconstruction and in 65.7% without reconstruction, after 24 months in 92.3% of cases with reconstruction and in 64.7% without reconstruction, after 36 months in 90% with reconstruction and in 72.3% without reconstruction. These differences were statistically very significant (p < 0.001).

At 48 months the continence with anterior and posterior reconstruction was preserved in 93.6% of cases, against 81% without reconstruction. This was also a statistically significant difference (p < 0.005).

At 60 months the preservation of continence with anterior and posterior reconstruction occurred in 97.6% of cases and without reconstruction in 90.3% of cases. This last is the only difference not statistically significant (p = 0.054).

### Limitations of the study

Despite several strengths our study has some limitations: Firstly the execution of the procedures with and without anterior and posterior reconstructions by two different surgeons. The lack of balance between the two groups (reconstruction vs no reconstruction); actually this represents a bias in reporting and interpreting data, but the patients were consecutive and, therefore, data were reported as pure. Lastly the populations that performed or not nerve sparing technique were not differentiated.

## Conclusions

In our case study with a long term follow-up the extraperitoneal RARP with anterior and posterior reconstructions technique seems to be associated with a higher rate of urinary continence and a faster recovery of the same, compared to the use of no reconstruction technique. This statistically significant advantage is appreciable in early continence, but remains so, although to a lesser extent, up to 48 months of follow-up.

## Data Availability

The datasets used and/or analyzed during the current study available from the corresponding author on reasonable request.
